# Quantitative oculomotor findings in migrainous patients

**Published:** 2014-10-06

**Authors:** Sara Momtaz, Fahimeh Hajiabolhassan, Mansoureh Togha, Shohre Jalaie, Amir Almasi

**Affiliations:** 1 Department of Audiology, School of Rehabilitation, Arak University of Medical Sciences, Arak, Iran; 2 Department of Audiology, School of Rehabilitation, Tehran University of Medical Sciences, Tehran, Iran; 3 Iranian Center of Neurological Research, Neuroscience Institute, Tehran University of Medical Sciences, Tehran, Iran; 4 Department of Biostatistics, School of Rehabilitation, Tehran University of Medical Sciences, Tehran, Iran; 5 Department of Epidemiology, School of Health, Arak University of Medical Sciences, Arak, Iran

**Keywords:** Videonystagmography, Oculomotor, Saccade, Smooth Pursuit, Optokinetic Nystagmus, Migraine

## Abstract

**Background:** Neurotologic signs and symptoms, especially vestibular symptoms are common in migrainous patients. Involvement of the visual system in migrainures has received a great deal of attention in recent years, but the oculomotor part of the visual system has been largely ignored. The goal of this study was to investigate some parts of the central vestibular system using the oculomotor part of videonystagmographic evaluation, including spontaneous nystagmus, gaze-evoked nystagmus, smooth pursuit, saccade and optokinetic ystagmus interictally in migrainous patients.

**Methods:** In this case–control study, 30 patients with migraine and 38 healthy volunteers within the age range of 18-48 years old were included spontaneous nystagmus; gaze-evoked nystagmus in right, left and up sides, smooth pursuit, optokinetic nystagmus using three different velocities and saccade test performed in both groups. The data were analyzed using SPSS for Windows 18.0.

**Results:** Some parameters of gain and phase and also morphology of the smooth pursuit, velocity of the saccade and slow phase velocity of optokinetic were significantly different in migrainures, although the statistical differences of these parameters were not clinically important as they were in the normal range of a defined device.

**Conclusion:** These results may suggest the presence of subtle otoneurologic abnormalities in migrainous patients that is probably due to the efficiency of oculomotor function with vestibulocerebellar origin.

## Introduction

Migraine is a common disorder with a prevalence of 15-17% in women and 5-8% in men.^1^

Involvement of the visual system in migrainous patients has received a great deal of attention in recent years, but the oculomotor part of the visual system has been largely ignored. This is surprising because recent genetic and imaging studies have shown that cerebellum that plays an important role in oculomotor control has been affected in patients who suffer from migraine.^2^

On the other hand, many migraine sufferers may experience a spectrum of balance disorders, which includes dizziness, constant imbalance and light-headedness that are not necessarily due to vestibular disturbances. These subjective feelings are extended to motion sickness, positional vertigo and episodic acute vertigo that have an undoubted vestibular origin.^3^ By such considering, we investigated migrainous patients by oculomotor tests with the aim of obtaining specific findings that may provide a clinical sign reflecting continuous dysfunction of cerebellar or brain stem structures.

## Materials and Methods

This was a case–control study in which 30 migrainous, 22 women and 8 men, enrolled using simple random sampling. The patients were recruited from April to December 2010 in the Audiology Department of the Rehabilitation Faculty of Tehran University of Medical Sciences, Tehran, Iran. This study was approved by the local Ethics Committee and patients were diagnosed by a neurologist according to International Headache Society criteria in 2004.^4^

Before the assessment, informed consent was obtained from all participants of this study. Oculomotor test was performed interictally after performing basic hearing evaluation (including pure tone air in 250-8000 per octave and immittance audiometry and also acoustic reflex). The results are reported in table 1.

The exclusion criteria included the presence of any kind of hearing loss, either conductive, mixed or sensory neural, any history of otologic disorders; presence of any kind of central nervous system disease; using tranquilizers and sedative drugs or tobacco or alcohol within 48 h before the test; using caffeinated drinks or chocolate on the day of examination; sleep deprivation on the night prior to the examination or presence of exhaustion during the exam; unwillingness to continue the test and being less than 18 or more than 48 years old.

Oculomotor test results were compared with 38 normal controls, 31 women and 7 men, with the same exclusion criteria. The age and sex difference between the two groups were not statistically different. All patients and normal individuals were evaluated by the same audiologist.

Oculomotor evaluation was performed by videonystagmography via Eye Dynamics VNG System, Canada.

Spontaneous nystagmus, gaze-evoked nystagmus and oculomotor evaluation (smooth pursuit, saccade and optokinetic) were performed for each subject. 

Statistical analysis was performed using SPSS for Windows 18.0 (SPSS Inc., Chicago, IL, USA) The Student’s t-test was used for continuous variables. All the tests were performed at α = 0.05 level of significance.

## Results

Thirty-eight normal subjects with the mean age of 30.15 ± 7.51 served as a control group; 81.60% of them were female. The average age of the patients was 35.80 ± 7.09 years and 73.30% of them were female. A history of migraine was present for 1-36 years (mean: 12.11 years). Number of headache days per month was from 1 day to all days of the month (mean 9.55/month). Visual Analog Scale (VAS) was used for pain severity estimation. In migraine patients, the VAS scale was 5-10 (mean = 8.07). 

Regarding smooth pursuit, gain of 0.2 Hz and phase of 0.4 Hz were statistically meaningful in the left eye between migrainous and normal subjects.

In the saccade test, left eye latency was the statistically meaningful parameter.

In the optokinetic test, the statistical difference parameter was gain and slow phase velocity (SPV) for 20°/s stimulus in both right and left eyes, while the stimulus was moving rightward.

**Table 1:** Smooth pursuit parameters (gain and phase), saccade parameters (velocity and latency), and optokinetic slow phase velocity comparison in three different speeds between migraine patients and normal group

**Test**	**Units**	**Right Eye**			**Left Eye**		
		**Migraine**	**Control**	**P**	**Migraine**	**Control**	**P**
Pursuit gain (%)	Velocity 0.1	110.85 (11.60)	111.68 (13.74)	NS	105.38 (10.15)	104.47 (11.08)	NS
	Velocity 0.2	105.07 (10.05)	102.26 (11.26)	0.008	102.14 (8.46)	96.79 (7.82)	0.009
	Velocity 0.4	101.63 (12.83)	97.76 (10.39)	NS	98.97 (10.93)	95.87 (11.05)	NS
Pursuit phase (°)	Velocity 0.1	-1.54 (2.56)	-1.24 (2.90)	NS	-1.77 (2.98)	-2.13 (3.94)	NS
	Velocity 0.2	-2.21 (3.49)	-3.29 (3.73)	NS	-1.38 (2.59)	-2.13 (3.25)	NS
	Velocity 0.4	-4.30 (4.45)	-3.63 (4.09)	NS	-0.97 (4.94)	-3.87 (4.76)	0.017
Saccade	Velocity(°/s)	512.9 (76.67)	529.76 (102.09)	NS	471.80 (75.06)	480.47 (77.10)	NS
	Latency (ms)	0.26 (0.059)	0.23 (0.03)	NS	0.28 (0.12)	0.25 (0.03)	0.008
OKN rightward	SPV 20 °/s	16.30 (2.86)	14.63 (3.04)	0.029	16.43 (3.73)	14.50 (3.21)	0.025
	SPV 30 °/s	19.90 (4.70)	14.6311 (3.20)	NS	19.93 (4.72)	18.86 (5.38)	NS
	SPV 40 °/s	20.17 (8.15)	19.05 (5.12)	NS	21.53 (8.85)	21.22 (8.55)	NS
OKN leftward	SPV 20 °/s	16.30 (3.06)	21.19 (8.75)	NS	16.37 (3.07)	14.97 (3.30)	NS
	SPV 30 °/s	18.83 (5.56)	15.47 (3.07)	NS	18.72 (5.65)	18.16 (5.91)	NS
	SPV 40 °/s	19.53 (8.76)	18.89 (5.35)	NS	18.80 (8.35)	20.72 (8.51)	NS

R: Right eye; L: Left eye; OKN: Optokinetic nystagmus; SPV: Slow phase velocity

## Discussion

Discrepancy in various studies regarding the occurrence of migraine and vestibular symptoms in association was the reason for performing this study.^5^

Less than 3° of spontaneous nystagmus can be considered within normal range. According to Von Brevern, spontaneous nystagmus with SPV <3° was insignificant. In our study, no pathological spontaneous and gaze-evoked nystagmus was found (SPV of >3°/s) in any of the migrainous patients interictally and was in consistency with other studies.^6^^,^^7^

Since oculomotor abnormalities have seen almost in the left eye, we may hypothesize that severity of damage was not that much robust, to be able to affect the dominant (right) eye. So this pathology may show subtle abnormalities that were observable in the recessive (left) eye in our study.

Studies have shown minor vestibular findings in the symptom-free phase of migraine.^6^ However, there are some reports of permanent vestibular disturbances in patients with migrainous vertigo that can be due to semicircular canal paresis, central vestibular problem^8^ and oculomotor abnormalities.^9^ Occurrence of similar (and often insignificant) abnormal vestibular test results and oculomotor abnormal findings has been reported in symptom-free intervals of migranous patients.^9^

Oculomotor findings in symptom-free intervals may reflect permanent neural disturbances in some brain stem pathways.

## Conclusion

The overall findings of this study show that using neurotologic investigation in migrainous patients may give us a little information on definite differential diagnosis of uncompensated, peripheral or central origin of their disturbances.

As our patients were tested interictally, questions remain regarding the effects of testing in and around the time of migrainous attacks. The effects of other factors such as the severity and frequency of symptoms, selecting a pure subtype of migraine, duration of illness along with other symptoms like anxiety and motion sickness may be worth ideas for more investigation.

## Notes

**How to cite this article: **Momtaz S, Hajiabolhassan F, Togha M, Jalaie Sh, Almasi A. Quantitative oculomotor findings in migrainous patients. Iran J Neurol 2014; 13(4): 250-2.
